# Io’s tidal response precludes a shallow magma ocean

**DOI:** 10.1038/s41586-024-08442-5

**Published:** 2024-12-12

**Authors:** R. S. Park, R. A. Jacobson, L. Gomez Casajus, F. Nimmo, A. I. Ermakov, J. T. Keane, W. B. McKinnon, D. J. Stevenson, R. Akiba, B. Idini, D. R. Buccino, A. Magnanini, M. Parisi, P. Tortora, M. Zannoni, A. Mura, D. Durante, L. Iess, J. E. P. Connerney, S. M. Levin, S. J. Bolton

**Affiliations:** 1 Jet Propulsion Laboratory, California Institute of Technology, Pasadena, CA USA; 2 Centro Interdipartimentale di Ricerca Industriale Aerospaziale, Alma Mater Studiorum – Università di Bologna, Forlì, Italy; 3 Department of Earth and Planetary Sciences, University of California, Santa Cruz, Santa Cruz, CA USA; 4 Department of Aeronautics and Astronautics, Stanford University, Stanford, CA USA; 5 Department of Earth, Environmental, and Planetary Sciences, Washington University, St. Louis, MO USA; 6 California Institute of Technology, Pasadena, CA USA; 7 Dipartimento di Ingegneria Industriale, Alma Mater Studiorum – Università di Bologna, Forlì, Italy; 8 Institute for Space Astrophysics and Planetology, National Institute for Astrophysics, Rome, Italy; 9 Sapienza Università di Roma, Rome, Italy; 10 NASA Goddard Space Flight Center, Greenbelt, MD USA; 11 Southwest Research Institute, San Antonio, TX USA

**Keywords:** Rings and moons, Planetary science

## Abstract

Io experiences tidal deformation as a result of its eccentric orbit around Jupiter, which provides a primary energy source for Io’s continuing volcanic activity and infrared emission^1^. The amount of tidal energy dissipated within Io is enormous and has been suggested to support the large-scale melting of its interior and the formation of a global subsurface magma ocean. If Io has a shallow global magma ocean, its tidal deformation would be much larger than in the case of a more rigid, mostly solid interior^2^. Here we report the measurement of Io’s tidal deformation, quantified by the gravitational tidal Love number *k*_2_, enabled by two recent flybys of the Juno spacecraft. By combining Juno^3,4^ and Galileo^5–7^ Doppler data from the NASA Deep Space Network and astrometric observations, we recover Re(*k*_2_) of 0.125 ± 0.047 (1*σ*) and the tidal dissipation parameter *Q* of 11.4 ± 3.6 (1*σ*). These measurements confirm that a shallow global magma ocean in Io does not exist and are consistent with Io having a mostly solid mantle^2^. Our results indicate that tidal forces do not universally create global magma oceans, which may be prevented from forming owing to rapid melt ascent, intrusion and eruption^8,9^, so even strong tidal heating—such as that expected on several known exoplanets and super-Earths^10^—may not guarantee the formation of magma oceans on moons or planetary bodies.

## Abstract (web summary)

By measuring the tidal deformation of Io as it orbits Jupiter using Juno Doppler and historically available data, the hypothesis of a shallow global magma ocean in Io is shown to be false.

## Main

Io is the innermost Galilean moon, orbiting Jupiter every 42.5 hours. It has a mean diameter of 3,643 km and a bulk density of 3,528 kg m^−3^, making it about 5% larger in both diameter and density than the Moon^7,11^. Owing to Io’s eccentric orbit, its distance from Jupiter varies by about 3,500 km, leading to variations in Jupiter’s gravitational pull. Similar to tides on the Moon raised by Earth, these gravitational variations cause tidal deformation on Io, which is theorized to serve as the primary energy source for the intense volcanic activity and infrared emission observed on Io’s surface^1,12^.

The amount of energy dissipated within Io is immense, with total power output around 100 TW (ref. ^13^). For decades, it has been speculated that this extreme tidal heating may be sufficient to melt a substantial fraction of Io’s interior, plausibly forming a global subsurface magma ocean. Many worlds are believed to have had magma oceans early in their evolution—notably the early Moon, which is thought to have had a shallow magma ocean in the first 100 Myr caused by the giant impact that birthed the body^14^. Io’s extreme volcanism strongly suggests the existence of at least a partially molten interior. Whether the interior contains a shallow global magma ocean has been an outstanding question since the discovery of Io’s volcanism^15^.

Melt is expected to migrate rapidly from such partially molten regions in the mantle^8,9,16^; whether it accumulates to form a magma ocean or simply erupts depends on many poorly understood properties, including the nature of the melt pathways, the melt volatile content and Io’s crustal density. Thus, there are two endmember models for Io’s interior: a partially molten but mostly solid interior or an interior with a global magma ocean. A metallic core is also indicated from earlier gravitational measurements and is probably liquid^7^.

The existence of a global magma ocean has been predicted by two types of analysis. Magnetic induction measurements from the Galileo mission suggested the existence of a magma ocean within Io and an approximately 50-km-thick near-surface layer with >20% melt^17^, although the results have been the subject of substantial debate^18–20^. Recently, the global mapping of Io’s volcanoes by Juno was used to suggest that the distribution of volcanic heat flow is consistent with the presence of a global magma ocean^21^, although there is recent debate about whether this technique can be used to distinguish whether Io’s volcanic activity is driven by a shallow global magma ocean^22^.

A measurement of Io’s tidal response is a key diagnostic for distinguishing whether Io has a global magma ocean or not. If Io does (not) have a magma ocean, the tidal response will be large (small)^2^. Io’s tidal response can be quantified by a complex number called the gravitational tidal Love number^23^ *k*_2_ = Re(*k*_2_) + iIm(*k*_2_). The real component Re(*k*_2_) characterizes the in-phase response, defined as the ratio of the imposed gravitational potential from Jupiter to the induced potential from the deformation of Io (Methods). The out-of-phase part of the tidal response Im(*k*_2_) is often defined as −|*k*|/*Q*, in which *Q* is the dissipation quality factor and is a measure of how much tidal heat Io should be generating. Previous studies have used astrometric measurements to determine |*k*_2_|/*Q*, but could not determine Re(*k*_2_) independently^24^.

## Measuring Io’s tidal response

**Extended Data Fig. 1:**
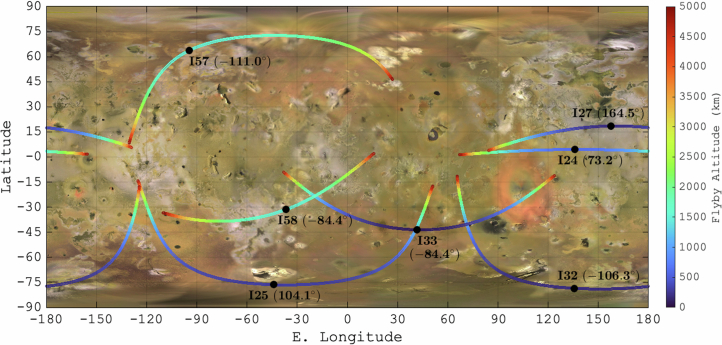
Ground tracks of Galileo (I24, I25, I27, I32 and I33) and Juno (I57 and I58) for altitude ≤ 5,000 km of the closest approach over a colour image mosaic of Io^52^. The altitudes were computed relative to a spherical Io, assuming an 1,829.4-km radius. The black circles represent the closest approaches. The angles in parentheses represent the true anomaly of Io with respect to Jupiter.

**Extended Data Fig. 2:**
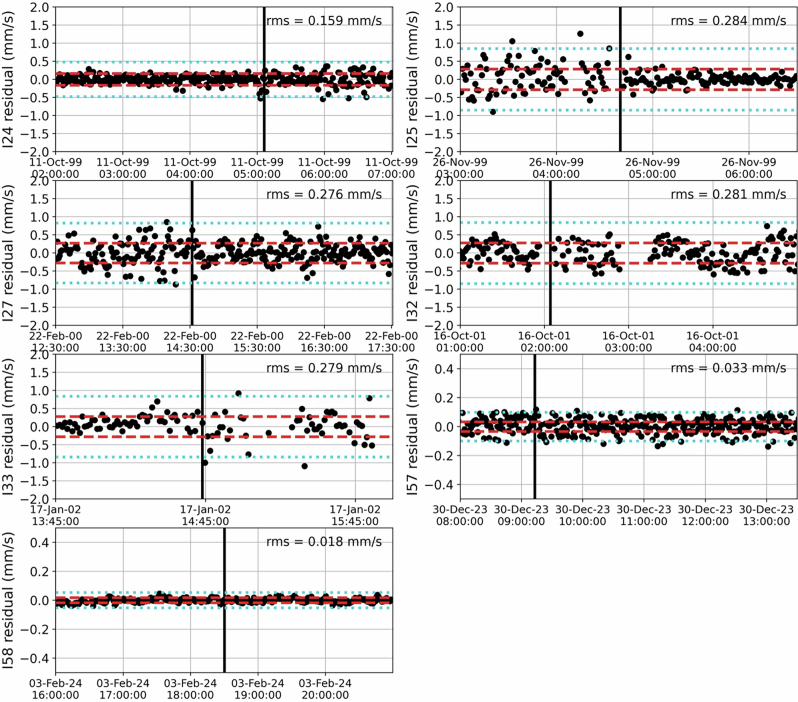
Doppler residuals of Galileo (I24, I25, I27, I32 and I33) and Juno (I57 and I58) near the closest approach to Io. The vertical black lines represent the time of the closest approach. The Doppler r.m.s. for each flyby is shown in the upper-right side of each plot. The red dashed and cyan dotted lines represent the ±1*σ* and ±3*σ* ranges of the Doppler r.m.s., respectively. Nearly all points are well within the 3*σ* range. The Juno Doppler data are generally about an order of magnitude more accurate than the Galileo Doppler data (note the scale of the axes).

The Juno spacecraft has been exploring the Jovian system since mid-2016^25^. By accurately tracking the motion of a spacecraft, the gravity field of a perturbing body can be recovered^26,27^. As of June 2024, Juno has completed a total of 62 orbits around Jupiter and the data acquired during this period have been used to improve our understanding of the dynamical environment at Jupiter, especially the orbits of the Galilean satellites and Jupiter’s gravity field and orientation^3,4,28,29^. The two flybys directly relevant for characterizing Io’s tidal response are denoted I57 and I58 and occurred on 30 December 2023 and 3 February 2024, respectively (Extended Data Fig. 1). I57 provided a unique opportunity to acquire the gravity data for Io’s high northern hemisphere. Two flybys of Io were designed as part of Juno’s extended mission to investigate and determine whether a global magma ocean exists in Io. Both flybys occurred at altitudes of about 1,500 km and provided close-proximity Doppler data, with an order of magnitude greater accuracy than the Galileo Doppler data (Extended Data Fig. 2). Combining the Juno data with the previously acquired Galileo data^7^ and astrometric observations^24^, we have recovered Re(*k*_2_) = 0.125 ± 0.047 (1*σ*) and *Q* = 11.4 ± 3.6 (1*σ*), yielding |*k*_2_|/*Q* = −Im(*k*_2_) = 0.0109 ± 0.0054 (1*σ*) (Extended Data Table 1). In our model, the tides in Jupiter resulting from Galilean satellites are assumed to have a constant time lag and our recovered estimate is 0.11693 ± 0.00069 s (1*σ*). Moreover, the recovered *J*_2_ and *C*_22_ for Io, including permanent tides, were (1,834.6 ± 1.5 × 10^6^ (1*σ*) and (549.6 ± 0.3 × 10^6^ (1*σ*), respectively, yielding a *C*_22_/*J*_2_ ratio of 0.2996 ± 0.0003 (1*σ*), consistent with the 0.3 expected for hydrostatic Io^5,7,30^.

## Tidal response modelling

**Fig. 1:**
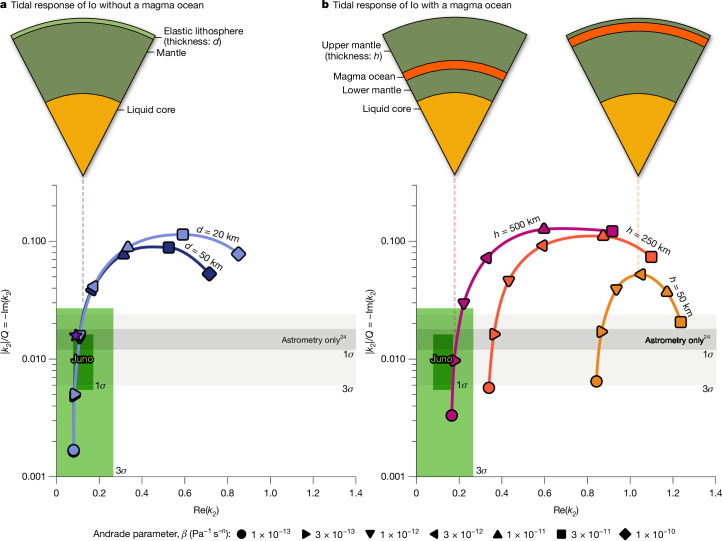
The measured tidal response (Re(*k*_2_) and |*k*_2_|/*Q*) of Io compared against models without and with a magma ocean. **a**, No magma ocean. Shaded green boxes are 1*σ* and 3*σ* Juno results (Methods) and shaded grey boxes are from a previous study based on astrometry^24^. Here a three-layer Io is assumed with an elastic lid of thickness *d*, a partially molten mantle with an Andrade parameter *β* (in Pa^−1^ s^−^^*n*^) as specified by the symbols and a liquid iron core. The second Andrade parameter is assumed to be *n* = 0.3. The purple star marker represents the model from Fig. 2 of ref. ^2^. **b**, The same as in **a** but for models including a magma ocean with upper mantle. Here the ocean is at a depth *h* and is sandwiched between two Andrade viscoelastic layers. The magma ocean is assumed to be 100 km thick. Increasing the upper-mantle thickness reduces Re(*k*_2_), as expected; to match the Juno results, the depth *h* exceeds 500 km, which correlates to a deep magma ocean. Further details are given in Methods.

Figure 1 compares the Juno measurements (shaded green box) with simple Io models both without (Fig. 1a) and with (Fig. 1b) a magma ocean (Extended Data Table 2). These models use a viscoelastic (Andrade) rheology in which the *β* parameter describes the amplitude of the anelastic deformation and is expected to be in the range 10^−13^–10^−^^10^ Pa^−1^ s^−^^*n*^ for partially molten silicates^31^ and *n* describes the time dependence of anelastic deformation. The effect of adding a magma ocean is most easily seen by comparing the two cases in which the elastic lid thickness (*d*; Fig. 1a) or upper-mantle thickness (*h*; Fig. 1b) is 50 km. Without a magma ocean, Re(*k*_2_) can be as small as about 0.1, at which point the measured |*k*_2_|/*Q* value is also satisfied; with a magma ocean, Re(*k*_2_) is never less than 0.8 when *h* = 50 km because the decoupling effect of the liquid layer leads to a larger tidal response. These results provide strong evidence demonstrating that a shallow global subsurface magma ocean capable of being the source of Io’s volcanic activity does not exist and are insensitive to the details of the rheology assumed because they arise from the mechanical decoupling effect of a liquid layer.

**Extended Data Fig. 3:**
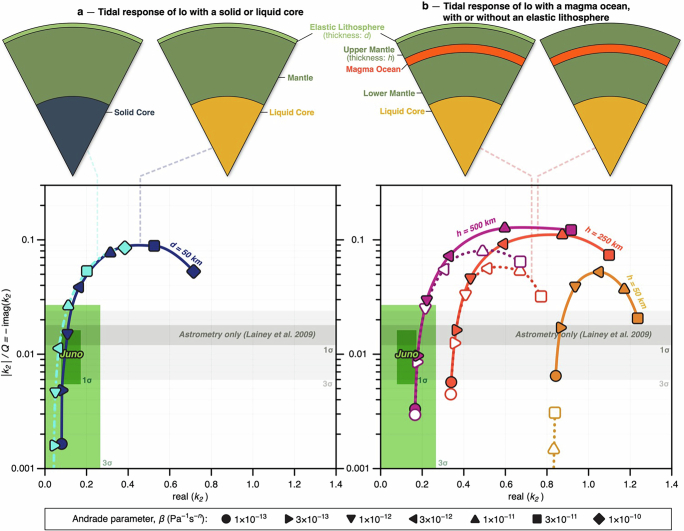
Effect of solid core and 50-km-thick elastic lid on tidal response. **a**, Effect of a solid core on the no magma ocean case. Solid and dashed lines represent cases with liquid and solid cores, respectively. The solid core case results in a lower Re(*k*_2_) for a given mantle rheology. **b**, Effect of adding a 50-km-thick elastic lid to the case with a magma ocean (that is, a five-layer case). Solid and dashed lines represent cases without and with an elastic lid, respectively.

**Extended Data Fig. 4:**
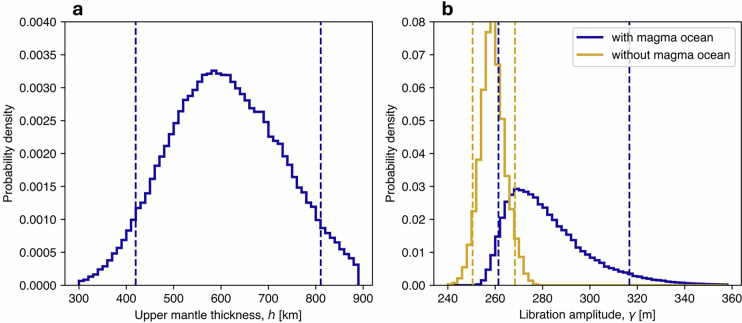
Posterior distribution of the upper mantle thickness and physical libration amplitude. **a**, Posterior distribution of the upper mantle thickness for the case with a magma ocean. The upper mantle thickness *h* represents the depth to the top of the global magma ocean layer. The median value of *h* is 605 km. The vertical dashed lines indicate 5th and 95th percentiles that correspond to thicknesses of 420 km and 810 km, respectively. At a 3*σ* level (0.135% probability), the lower bound on the upper mantle thickness is 318 km. **b**, Posterior distribution of the physical libration amplitude for the cases with and without a magma ocean. The with magma ocean case represents the distribution of solutions with a magma ocean, whose depth is constrained by the observed static gravity and Love numbers. Although the two probability distributions overlap, smaller libration amplitudes are possible for the no magma ocean case, indicating that future measurements of libration could exclude the deep magma ocean case.

**Extended Data Fig. 5:**
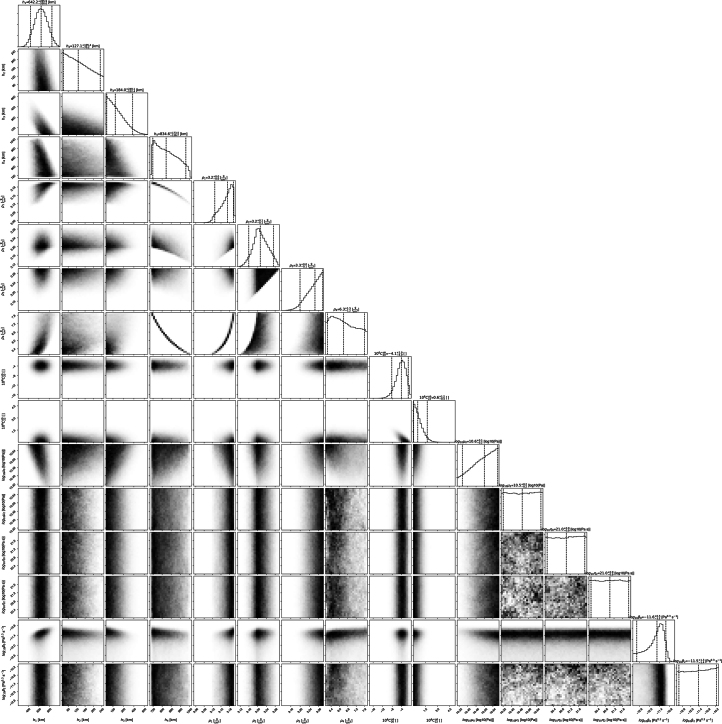
Corner plot showing the posterior distribution of Io’s internal structure parameters for the case with a magma ocean. The variables are as follows: *h*_*i*_ are layer thicknesses, *ρ*_*i*_ are layer densities, *δC*_*nm*_ are non-hydrostatic contributions to gravity coefficients *C*_*nm*_, *μ*_*i*_ are shear moduli, *η*_*i*_ are viscosities and *β*_*i*_ are the Andrade rheology parameters. The layers are numbered from the outermost layer inward.

**Extended Data Fig. 6:**
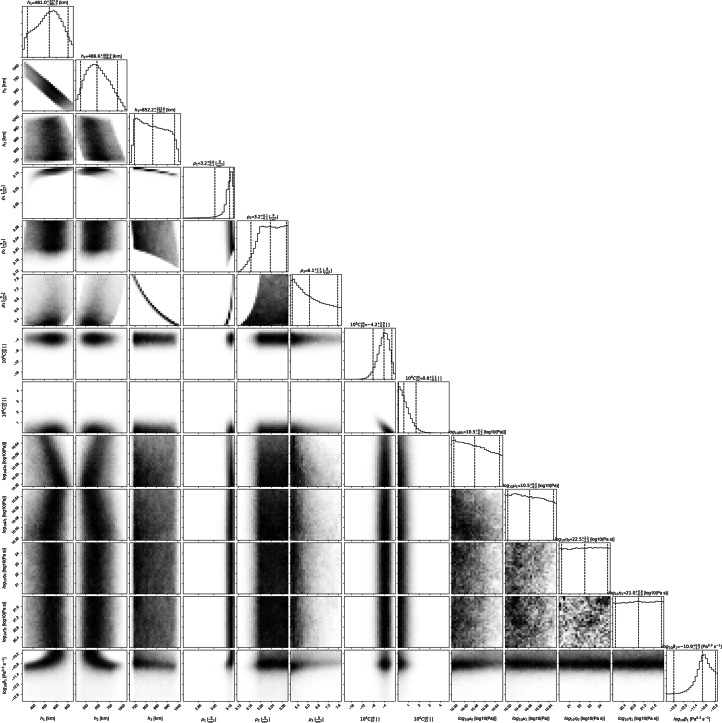
Corner plot showing the posterior distribution of Io’s internal structure parameters for the case without a magma ocean. The variables are as follows: *h*_*i*_ are layer thicknesses, *ρ*_*i*_ are layer densities, *δC*_*nm*_ are non-hydrostatic contributions to gravity coefficients *C*_*nm*_, *μ*_*i*_ are shear moduli, *η*_*i*_ are viscosities and *β*_*i*_ are the Andrade rheology parameters. The layers are numbered from the outermost layer inward.

A thicker viscoelastic upper mantle overlying the magma ocean will reduce the surface deformation. Figure 1b shows that an upper mantle 250 km thick (orange line) reduces Re(*k*_2_) but not by enough to satisfy the Juno measurement. However, an upper mantle with a thickness of approximately 500 km (purple line) can reproduce the measured Re(*k*_2_) and |*k*_2_|/*Q*. We confirm this result by conducting a comprehensive Markov chain Monte Carlo (MCMC) study of Io’s internal structure using *k*_2_ and degree-2 gravity coefficients (Extended Data Table 1) as observations for the cases with and without a magma ocean (Methods). Our full model input is given in Extended Data Table 3 for the magma ocean case and Extended Data Table 4 for the no magma ocean case. For the case with a magma ocean beneath a viscoelastic (Andrade) mantle, our result shows that the thickness of the mantle must be greater than 318 km (at a 3*σ* level; Extended Data Fig. 4a). Full posterior distributions with and without a magma ocean are shown in Extended Data Figs. 5 and 6, respectively. The Juno results do not exclude the possibility of a deep magma ocean existing at a depth of >318 km, although a deep magma ocean could not be the source of Io’s volcanic activity and we suggest such a deep magma ocean would resemble more the proposed basal magma ocean on Earth^32^, and perhaps Mars^33^, rather than a shallow, Moon-like magma ocean^34^. Also, adding a surface elastic layer to the magma-ocean-bearing models does not change our conclusions (Extended Data Fig. 3b).

**Fig. 2:**
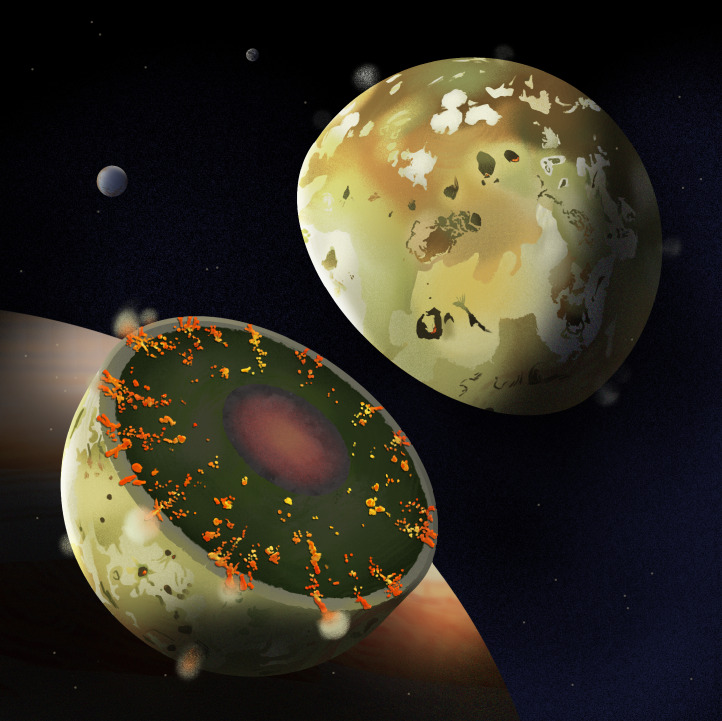
The internal structure of Io as revealed by the present study. Our estimate of *k*_2_ suggests that Io does not have a shallow global magma ocean and is consistent with that expected for a mostly solid mantle (green hues), with substantial melt (yellows and oranges), overlying a liquid core (red/black). Artist rendering by Sofia Shen (JPL/Caltech).

A very thin (<2 km), shallow magma ocean might produce a small Re(*k*_2_) consistent with our observations^35^. However, Io’s long-wavelength surface topography has amplitudes of about 1 km (ref. ^36^) and isostatic variations in the lid thickness will result in basal topography of at least a few kilometres, depending on the density contrast. We suggest that, for a very thin magma ocean, grounding would probably occur, and the magma ocean would no longer be global. We conclude that a shallow, global magma ocean is excluded by the Juno results and Fig. 2 presents an artistic illustration of Io’s interior based on our results.

Because a deep global magma ocean is expected to mechanically decouple the crust, we explore the potential for measurements of diurnal librations of the surface to provide further constraints. Our MCMC analysis (Methods) shows that the posterior probability distributions of the libration amplitudes for cases with and without a magma ocean have a substantial overlap (Extended Data Fig. 4b). For a no magma ocean case, the libration amplitude ranges from 250 m to 268 m (5–95% confidence interval). For the magma ocean scenario, the libration amplitude could be larger, ranging from 261 m to 317 m. Both values are at the lower end of the past predictions^37^ owing to the observed low value of Re(*k*_2_) from this study that requires a thick outer shell.

Magnetic induction has been suggested as another method to determine whether Io has a global magma ocean^17^. However, detecting a deep magma ocean using a magnetic induction technique may be challenging because of saturation at a relatively low melt fraction^38^. The geometric tidal Love number, *h*_2,_ also provides constraints on the tidal response; however, similar to *k*_2_, we expect that this measurement would also be unable to discriminate between basal magma ocean and no magma ocean cases. Other measurements, such as obliquity, precession, nutation and high-resolution gravity field, could also contribute to investigating Io’s deeper interior.

## Io’s lack of a shallow magma ocean

Our results indicate that a shallow global magma ocean in Io does not exist, and these findings are supported by our present knowledge of Io’s long-wavelength shape^39^. On Earth, deep melts can be denser than the surrounding mantle and thus remain sequestered in a basal magma ocean^40^. On Io, pressures are much lower, so mantle melts are expected to be always less dense than the surrounding solid mantle. The melts will tend to ascend, making maintenance of a deep magma ocean dynamically problematic. Conversely, if the melts are dense (for example, if sufficiently iron-rich), although a deep magma ocean could then form, it would be hard to explain how any such melt would ascend and erupt. Thus, we conclude that the volcanism seen on Io’s surface is not sourced from a global magma ocean. Although we cannot exclude a heterogeneous mantle^41^ in which both deep, dense melts and buoyant erupting magmas are present, no observations so far support the existence of a deep molten layer.

How did the early Moon retain a shallow magma ocean for a relatively extended period^14^, whereas Io, which is continually tidally heated, does not? Two possibilities are a relative absence of volatiles on the Moon to drive eruptions or the presence of the low-density anorthositic crust, which impedes upwards melt migration and eruption^42^. Although Io’s crustal thickness and structure are uncertain^43,44^, volatile-driven eruptions are common^45^. The Moon’s magma ocean originated as a result of its formation by a giant impact; in the absence of such a catastrophic event, tidal heating alone seems insufficient to allow such a magma ocean to develop at Io.

Understanding tidal heating is important as a primary cause of oceans within our Solar System, such as those on Europa and Enceladus^46^ and potentially beyond. Although it is commonly assumed among the exoplanet community that intense tidal heating may lead to magma oceans^10,47–49^, the example of Io shows that this need not be the case. Arguments that imply that Vesta or other very early accreted asteroids or asteroidal parent bodies formed magma oceans from ^26^Al decay heating may also need to be re-examined^50,51^. Rapid melt migration and eruption may frustrate the development of magma oceans^8^, unless there exists a barrier to upward motion. Such barriers probably existed for the early Moon and also for icy satellites, for which the ‘melt’ (water) is denser than the ‘crust’ (ice) and oceans are common^46^. Neither intense surficial silicate volcanism nor extreme tidal heating necessarily imply a shallow magma ocean.

## Methods

### Dataset

The dataset used in this study includes the Deep Space Network radiometric data acquired during the Io flybys of the Juno^3,4^ and Galileo^5–7^ spacecraft, as well as astrometric observations^24^. The primary Juno data consist of simultaneous two-way X-band (8.4 GHz) and Ka-band (32 GHz) data, referenced to X-band uplink, during I57 (30 December 2023) and I58 (3 February 2024). Both flybys occurred at an altitude of approximately 1,500 km with a relative velocity of about 30 km s^−1^. I57 was the only close approach in the high northern hemisphere, which was particularly helpful for acquiring improved global coverage of gravity data.

The primary Galileo data consist of the S-band (2.3 GHz) two-way Doppler data acquired during five flybys: I24, I25, I27, I32 and I33 at lower signal-to-noise ratio than the measurements of Juno owing to the Galileo high gain antenna deployment anomaly. Details on Galileo flybys can be found in ref. ^7^ and discussions on the astrometric dataset used in this study can be found in ref. ^24^. The ground tracks and flyby altitudes of Galileo and Juno are shown in Extended Data Fig. 1 for altitude ≤5,000 km of the closest approach over a colour image mosaic of Io^52^. The flybys sample different true anomalies, latitudes and longitudes, providing good coverage for measuring the long-wavelength gravitational signature of Io.

### Data calibration

The Doppler data from the Deep Space Network and spacecraft are affected by the media in between. The Earth troposphere and ionosphere effects are calibrated using a standard method^53^. Furthermore, the Doppler data were calibrated for the path delay resulting from the Io plasma torus (IPT), a region of plasma generated by the ionization of the particles ejected by Io’s volcanic activity^54^.

For Juno I57 and I58 data, using the dual-frequency X-band and Ka-band data with the primary dataset being X-up/X-down and X-up/Ka-down during the closest approach, the IPT path delay owing to dispersive sources was calibrated using the dual-frequency downlink data. This calibration allowed for the direct extraction of the downlink leg Doppler shift caused by dispersive media^55,56^. Then, the contribution on the uplink leg was corrected by scaling the actual downlink contribution to account for the different uplink carrier frequency (7.1 GHz).

The Galileo high gain antenna failed to completely open, markedly reducing the signal-to-noise ratio for the Doppler tracking. The Galileo Doppler noise was dominated by instrumental noise rather than the expected interplanetary plasma noise. To calibrate Galileo Doppler data, the total electron density of the IPT has been integrated along the line of sight of the spacecraft using parametric models of the electron density distribution in the Jovian environment. The accuracy of these models is limited by the spatial and temporal variability of the IPT^57,58^. We built electron density distribution models for the warm torus for each Galileo flyby using the data acquired by the Plasma Wave Subsystem (PWS)^59^ during the same flyby, thus, using direct information about the electron density of the IPT at the moment of the Doppler measurements. The local electron densities of the plasma extracted from the PWS data were projected into the centrifugal equator along the magnetic field lines of the dipole model using a scale height function of the centrifugal equator distance and assuming longitudinal symmetry. Subsequently, following ref. ^60^, the electron density was fitted with Gaussian functions. Because the only Galileo flyby of Io that acquired PWS observations of the cold and ribbon tori was I00, during the Jupiter orbital insertion, two different models were generated for each flyby. One used the cold and ribbon observations from I00 Galileo flyby and was applied in I24, I25 and I27. The other used the cold torus and ribbon shapes from ref. ^60^, derived from Voyager data, and it was applied in I32 and I33. The choice of the model was decided by evaluating its performance for each flyby. Finally, the expected Doppler shift has been derived from the computed path delay and used to calibrate the data. The use of IPT-calibrated observables resulted in roughly a factor of two improvement in the root mean square (r.m.s.) of the residuals.

The Doppler residuals of Galileo (I24, I25, I27, I32 and I33) and Juno (I57 and I58) are shown in Extended Data Fig. 2. Note that the I57 noise was dominated by Earth’s troposphere noise, whereas the I58 noise was dominated by plasma interactions. In general, we weigh the data per Deep Space Network pass and the data weights are further refined on the basis of various simulations to ensure that our weighting scheme is robust. We note that some of the data points show non-Gaussian behaviour, but we have a large enough dataset to still perform a least-squares fit and rely on the central limit theorem when interpreting the statistics. One key point to note is that our results do not vary in a statistically significant way even if we remove the residual points exceeding or near the 3*σ* level.

### The gravity field of Io

The gravitational potential, *U*(*r*, *λ*, *ϕ*), associated with Io is expressed as a spherical harmonic expansion^27,61,62^:

1 $$U(r,\lambda ,\phi )=\frac{{\mu }_{{\rm{i}}}}{r}\mathop{\sum }\limits_{l=0}^{\infty }\mathop{\sum }\limits_{m=0}^{l}{\left(\frac{R}{r}\right)}^{l}{P}_{lm}(\sin \phi )[{C}_{lm}\cos (m\lambda )+{S}_{lm}\sin (m\lambda )]$$

in which *μ*_i_ is the mass parameter of Io, *l* is the spherical harmonic degree, *m* is the order, *P*_*lm*_ are the unnormalized associated Legendre polynomials, *C*_*lm*_ and *S*_*lm*_ are the unnormalized spherical harmonic coefficients, *R* is the reference radius of Io (1,829.4 km), *λ* is longitude, *ϕ* is latitude and *r* is the distance. The spherical coordinates (*λ*, *ϕ*, *r*) are evaluated at the spacecraft position relative to the Io body-fixed frame. In this formulation, zonal coefficients are defined as *J*_*l*_ = −*C*_*l*0_. The gravity field is modelled in an Io-body-fixed frame, in which the body pole direction is aligned with its orbit-normal direction and the body *x* axis is pointed along the Io–Jupiter direction at the periapsis. Io is in synchronous rotation, in which the period of rotation matches the orbital period. As we are assuming that the origin of the Io body-fixed frame is defined to be Io’s centre of mass, the degree-1 coefficients are identically zero.

The effects of the tide raised on Io by Jupiter can be modelled as corrections to Io’s gravitational harmonic coefficients^63–66^:

2 $${\Delta J}_{2}=| {k}_{2}| \left(\frac{{\mu }_{{\rm{j}}}}{{\mu }_{{\rm{i}}}}\right){\left(\frac{R}{{r}_{{\rm{ij}}}}\right)}^{3}\left(\frac{1-3{\sin }^{2}{\phi }_{{\rm{j}}}}{2}\right)$$

3 $${\Delta C}_{21}-{\rm{i}}{\Delta S}_{21}=| {k}_{2}| \left(\frac{{\mu }_{{\rm{j}}}}{{\mu }_{{\rm{i}}}}\right){\left(\frac{R}{{r}_{{\rm{ij}}}}\right)}^{3}(\sin {\phi }_{{\rm{j}}}\cos {\phi }_{{\rm{j}}}){{\rm{e}}}^{-{\rm{i}}({\lambda }_{{\rm{j}}}+\delta )}$$

4 $${\Delta C}_{22}-{\rm{i}}{\Delta S}_{22}=| {k}_{2}| \left(\frac{{\mu }_{{\rm{j}}}}{{\mu }_{{\rm{i}}}}\right){\left(\frac{R}{{r}_{{\rm{ij}}}}\right)}^{3}\left(\frac{1-{\sin }^{2}{\phi }_{{\rm{j}}}}{4}\right){{\rm{e}}}^{-2{\rm{i}}({\lambda }_{{\rm{j}}}+\delta )}$$

in which *k*_2_ represents the degree-2 gravitational tidal Love number, *μ*_j_ represents the mass parameter of Jupiter, *r*_ij_ represents the distance from Io to Jupiter, *δ* represents the tidal lag angle and *λ*_j_ and *ϕ*_j_ represent the Io-fixed longitude and latitude of Jupiter, respectively. These corrections vary with time as Io moves around Jupiter, causing periodic variations in *λ*_j_, *ϕ*_j_ and *r*_ij_. It is important to note that the corrections have non-zero average values known as the ‘permanent tide’. Extended Data Table 1 includes the permanent tide values determined on the basis of our estimated Re(*k*_2_) = 0.125 by averaging Δ*J*_2_ and Δ*C*_22_ over the Galileo to Juno time period.

The determination of Io’s gravitational coefficient, *C*_22_, was made in 1996^6^. Because of the limited data from the single flyby, only the single coefficient could be determined. Consequently, the hydrostatic equilibrium constraint was imposed by forcing *J*_2_ to be exactly 10/3 of *C*_22_. After the first Io flyby of the Galileo Millennium Mission (GMM), Io’s gravitational quadrupole moments (second degree and order gravitational harmonics) were recovered from the data acquired during four flybys of the prime mission, Galileo Europa Mission and GMM^5^. The dataset was sufficiently robust that the hydrostatic constraint was not needed and omitted. After the completion of the GMM, we extended the gravity analysis by adding the data from the final Io flyby^7^.

As with the previous published analyses, we found that there is no notable sensitivity in the dataset to the gravity field of degree higher than the quadrupole. Extended Data Table 1 shows our gravity results, along with those previously published. The *C*_21_ and *S*_21_ are related to the misalignment of the satellite’s principal axes and body coordinate axes. Their small values confirm that the two systems are nearly aligned. The small *S*_22_ value is primarily a consequence of the principal axis prime meridian not completely matching our coordinate system prime meridian as defined by the subplanet direction. We find the ratio of our total *C*_22_ to our total *J*_2_ is 0.2996 ± 0.0003 (1*σ*), nearly the 0.3 required for hydrostatic equilibrium^30^. A truly hydrostatic (fluid) Io could be subject to a slightly non-synchronous (or pseudo-synchronous) rotation owing to the non-zero orbital average of the diurnal tidal torque^67^. The small *S*_22_ value, with the uncertainty consistent with zero, aligns with an offsetting torque owing to a permanent (or quasi-permanent, that is, geologically ephemeral) non-hydrostatic mass distribution. This distribution stabilizes Io in the 1:1 spin–orbit resonance, similar to what is observed for Earth’s Moon^68^.

### Effect of Io’s libration

The expected amplitude of Io’s diurnal libration is about 275 m in the absence of a magma ocean^37^. Although with a magma ocean the diurnal libration of the crust can be much larger than these values, the detectability through radiometric data is limited to its solid interior, which should have low values^37^. We implemented a libration model by imposing Io’s prime meridian to point the instantaneous perifocus of its orbit^69,70^. Then, the forced physical libration at the orbital period is modelled as *γ* = *A*sin*M*, in which *M* represents the mean anomaly and *A* is the amplitude of the physical libration. Because the available data are not sensitive enough to detect the diurnal libration of Io, we assessed its effect in our analysis including different amplitudes of libration ranging from 10 to 500 m. In all cases, the estimated *k*_2_ remained within 1*σ* of its nominal value, indicating that the recovery of *k*_2_ is insensitive to Io’s libration at the accuracy level of the recovered quadrupole moments.

### The tides in the Jovian system

Tidal interaction is presumed to play a crucial role in the long-term evolution of the orbits of the Galilean satellites. Io’s active volcanism and associated heat flow are driven by tidal dissipation within the satellite. It is of great interest to determine whether Io is spiralling into or away from Jupiter. If the former is true, Io is losing more energy through internal dissipation than it is gaining from the torque on the tidal bulge that it raises on Jupiter. The amount of heat produced by tidal friction has a direct bearing on the thickness of its outer shell or lithosphere and the nature of the internal melt distribution, including the possibility of a subsurface magma ocean.

The tide model is based on the theory of equilibrium tides in which the gravitational attraction of a point mass distorts a spherical body, resulting in a tidal bulge. The acceleration acting on body a as a result of a tidal bulge raised by body b on Jupiter is^24,71^:

5 $${{\bf{a}}}_{{\rm{a}}}=\frac{3}{2}{\mu }_{{\rm{b}}}\frac{| {k}_{2}^{{\rm{j}}}| }{{R}_{{\rm{j}}}^{2}}{\left(\frac{{R}_{{\rm{j}}}}{{r}_{{\rm{aj}}}}\right)}^{4}{\left(\frac{{R}_{{\rm{j}}}}{{r}_{{\rm{bj}}}^{* }}\right)}^{3}\{[1-5{({\widehat{{\bf{r}}}}_{{\rm{aj}}}\cdot {\widehat{{\bf{r}}}}_{{\rm{bj}}}^{* })}^{2}]{\widehat{{\bf{r}}}}_{{\rm{aj}}}+2({\widehat{{\bf{r}}}}_{{\rm{aj}}}\cdot {\widehat{{\bf{r}}}}_{{\rm{bj}}}^{* }){\widehat{{\bf{r}}}}_{{\rm{bj}}}^{* }\}$$

in which *μ*_b_ is the mass parameters (that is, *GM*) of body b, ${k}_{2}^{{\rm{j}}}$ is the Love number of Jupiter, *R*_j_ is the Jupiter radius, *r*_aj_ and ${r}_{{\rm{bj}}}^{* }$ are the respective distances between Jupiter and bodies a and b and ${\widehat{{\bf{r}}}}_{{\rm{aj}}}$ and ${\widehat{{\bf{r}}}}_{{\rm{bj}}}^{* }$ are the respective directions from Jupiter to bodies a and b. Because Jupiter does not respond instantaneously to tide raising body b, the tidal bulge is offset from its present direction. We introduce this offset by assuming that there is simply a time delay Δ*t*_b_ between when the tidal bulge is raised and when it acts on body a. Consequently, the relation between position vector ${{\bf{r}}}_{{\rm{bj}}}^{* }$ and the present position *r*_bj_ is:

6 $${{\bf{r}}}_{{\rm{bj}}}^{* }={{\bf{r}}}_{{\rm{bj}}}-{\Delta t}_{{\rm{b}}}\,({\dot{{\bf{r}}}}_{{\rm{bj}}}+{\dot{W}}_{{\rm{j}}}\,({{\bf{r}}}_{{\rm{bj}}}\times {\widehat{{\bf{h}}}}_{{\rm{j}}}))$$

in which ${\dot{{\bf{r}}}}_{{\rm{bj}}}$ is the velocity of body b relative to Jupiter, ${\dot{W}}_{{\rm{j}}}$ is the rotation rate of Jupiter and ${\widehat{{\bf{h}}}}_{{\rm{j}}}$ is Jupiter’s pole direction. More details can be found in previous studies^23,24,72^.

The tidal time delay is related to the measure of the tidal dissipation, the tidal quality factor *Q*_b_, through the lag angle *δ*_b_:

7 $${\delta }_{{\rm{b}}}=\frac{{\Delta t}_{{\rm{b}}}}{{r}_{{\rm{bj}}}}\,|{\dot{{\bf{r}}}}_{{\rm{bj}}}+{\dot{W}}_{{\rm{j}}}({{\bf{r}}}_{{\rm{bj}}}\times {\widehat{{\bf{h}}}}_{{\rm{j}}})|$$

The quality factor is related to the lag angle (*δ*_b_) by ${Q}_{{\rm{b}}}^{-1}=\tan 2{\delta }_{{\rm{b}}}.$ The relationship between the quality factor *Q* and *k*_2_ is often represented as Im(*k*_2_) = −|*k*_2_|/*Q*.

For the case of a tide raised on a synchronously rotating satellite c by Jupiter, we consider only the self-tide (the effect on the satellite by the tide raised on it). We assume that the satellite pole is aligned with the orbit normal, ${\widehat{{\bf{h}}}}_{{\rm{c}}}$. Consequently, the acceleration as a result of the tide raised on a synchronously rotating satellite is:

8 $${{\boldsymbol{a}}}_{{\rm{c}}}=-\,3\,| {k}_{2}^{{\rm{c}}}| \,\left(\frac{{\mu }_{{\rm{j}}}}{{R}_{{\rm{c}}}^{2}}\right){\left(\frac{{R}_{{\rm{c}}}}{{r}_{{\rm{jc}}}}\right)}^{7}\left\{\left[1+3\Delta {t}_{{\rm{c}}}\frac{({\widehat{{\bf{r}}}}_{{\rm{jc}}}\cdot {\dot{{\bf{r}}}}_{{\rm{jc}}})}{{r}_{{\rm{jc}}}}\right]{\widehat{{\bf{r}}}}_{{\rm{jc}}}+\Delta {t}_{{\rm{c}}}({n}_{{\rm{c}}}-{\dot{\theta }}_{{\rm{c}}})({\widehat{{\bf{r}}}}_{{\rm{jc}}}\times {\widehat{{\bf{h}}}}_{{\rm{c}}})\right\}$$

in which ${\dot{\theta }}_{{\rm{c}}}$ is the satellite’s instantaneous angular velocity and the satellite’s rotation rate matches its mean orbital motion, that is, ${\dot{W}}_{{\rm{c}}}={n}_{{\rm{c}}}$. Specifically, we use the average rotation rate over the 100 years from 1950 to 2050 for the rotation rate, ${\dot{W}}_{{\rm{c}}}$. The angular velocity is computed as the magnitude of the orbital angular momentum divided by the square of the radial distance. The assumption is that the rotation rate will not vary substantially over several hundred years, periodic variations owing to librations are small and the tidal torque has nearly damped out. The delayed tidal force has a radial component proportional to the radial velocity and a component perpendicular to the radial direction and proportional to the difference between the mean motion and the instantaneous angular velocity. By convention, for satellites, we compute the quality factor *Q*_c_ from the lag angle ${\delta }_{{\rm{c}}}=\frac{1}{2}{n}_{{\rm{c}}}\Delta {t}_{{\rm{c}}}.$ The gravitational effects of the bulges raised on Jupiter tend to move the satellites away from Jupiter, decreasing their mean motions. The tidal bulge raised by Jupiter on Io has the opposite effect.

We numerically integrated the orbits of Galilean satellites with tide models and fit them to the spacecraft and astrometry data. The models and estimated parameters for the Jupiter satellite ephemeris and gravity field are broadly similar to the work done on the Saturnian system^73^. The positions and masses of the Sun, Moon and planets are from the JPL planetary ephemeris DE440 (ref. ^74^). Our estimated tidal time lag of Io is Δ*t* = 2,129.6 ± 677.0 s (1*σ*), which corresponds to *Q* = 11.4 ± 3.6 (1*σ*). Combining *k*_2_ and *Q*, we get *k*_2_/*Q* = −Im(*k*_2_) = 0.0109 ± 0.0054 (1*σ*), which is consistent with *k*_2_/*Q* = 0.015 ± 0.003 (1*σ*) from ref. ^24^. We note that, because Io’s rotation rate is known with much higher accuracy, any meaningful error in the rotation rate, both secular and periodic, would have a minimal effect within the uncertainty of the recovered Δ*t*. In fact, a first-order analysis suggests that if forced libration with an upper-end amplitude of the constraint based on our *k*_2_ estimate were to exist (Extended Data Fig. 4), the resulting error in the time delay would only be at a few percent level, which is substantially below the accuracy of the recovered tidal delay. Thus, although in theory it may be possible that the small angle effect would potentially show up for a very long period, it is not important for the relatively short time span considered in our study. Furthermore, in our model, we assume the tides in Jupiter have a constant time lag. Our recovered estimate is 0.11693 ± 0.00069 s (1*σ*), which corresponds to *Q*_jupiter_ = 31,733 ± 188 (1*σ*) at the Io frequency and is consistent with previous results^24^. We also note that, although *k*_2_ is primarily determined from Juno and Galileo data, the *Q* values for both Io and Jupiter are primarily determined from the long-term dynamics of the Galilean satellites by means of ground-based astrometry. In other words, *k*_2_ and *Q* are independently estimated and are not correlated.

### Interior modelling of Io

Because of the large uncertainties in appropriate parameters to use, we use a simplified model for Io’s structure. A more complicated, self-consistent approach^2^ yields essentially identical results (purple star marker in Fig. 1a). On the basis of Io’s bulk density and moment of inertia, we assume an iron/iron sulfide core to have a radius of 950 km and a density of 5,150 kg m^−^^3^ and the mantle to have a density of 3,259 kg m^−^^3^ and an outer radius of 1,820 km (ref. ^7^) (Extended Data Table 2). The core is assumed to be liquid and the mantle to have an infinite-frequency shear modulus of 40 GPa, which is at the upper end of that expected for partially molten olivine. Lower shear moduli would make it more challenging to match the measured *k*_2_ with a magma ocean. Note that, although constrained by static gravity field observations, there is some uncertainty about the composition and size of the metallic core—which we do not explore here. But these uncertainties will have a small effect compared with the state of the core (solid or liquid). Extended Data Fig. 3a shows that the difference between a solid and liquid core is small; a solid core reduces both Re(*k*_2_) and *k*_2_/*Q* for the same mantle rheology (and the change for a partially liquid core would be smaller still). Also, we note that a silicate mantle at or above the solidus will exceed the melting point of any plausible Fe–FeS core composition at core pressures^7^. Our baseline models assume a 100-km-thick magma ocean (see below).

Our three-layer Io has a purely elastic lid (with thickness *d*) with a single viscoelastic mantle layer beneath, consistent with expectations that, for a heat-pipe body such as Io, there will be a cold and rigid near-surface layer^75^. The four-layer model has two viscoelastic layers, separated by a magma ocean (Fig. 1). A purely elastic (as opposed to viscoelastic) top layer would reduce |*k*_2_|/*Q* to well below the measured value. The effect of adding a 50-km-thick elastic lid to the magma ocean case (that is, a five-layer model) is shown in Extended Data Fig. 3b. The effect is negligible at low Re(*k*_2_) values because, in these cases, the primary resistance to tidal deformation is because of the mantle and not the lid.

The viscoelastic mantle is described by a single Andrade rheology, details of which may be found in ref. ^2^. We do not use a Maxwell model as it provides a poor description of the rheology of real geological materials^31^. We assume that the Andrade parameter *n* = 0.3 throughout and vary the *β* parameter as noted in Fig. 1. We take the mantle viscosity to be 10^21^ Pa s, but varying this value does not affect our results unless the viscosity chosen is <10^15^ Pa s. In the Andrade model, the effective forcing frequency is related to the actual forcing frequency through an Arrhenius term that accounts for the changing response as a function of temperature^2^. We take this term to be 3.16, representing mantle material that is close to the melting point.

Our baseline models all assumed a magma ocean thickness of 100 km and varied its depth. We also investigated the effect of reducing the magma ocean thickness and found that a magma ocean that is 5 km and 2 km thick resulted in reductions in Re(*k*_2_) of 0.3% and 7.7%, respectively. It is noted that all our models neglect inertial terms and thus neglect the more complicated dynamics treated in refs. ^35,76^; in common with most models, they also neglect bulk dissipation^77^.

For a given internal model of Io, the complex Love number *k*_2_ is computed for the tidal response of a viscoelastic body composed of solid and liquid layers^78,79^. The forced libration amplitude of a given internal model of Io is computed using an approach that includes viscoelastic Andrade rheology^80^. The response of a viscoelastic layer relative to a fluid response is described by the layer-wise tidal and fluid Love numbers, ${k}_{2}^{{\rm{j}}}$ and ${k}_{2,{\rm{f}}}^{{\rm{j}}}$, respectively. The fluid Love number ${k}_{2,{\rm{f}}}^{{\rm{j}}}$ describes a layer within a body in hydrostatic equilibrium, found from the flattening factors computed for a multilayered Io^30^.

### MCMC internal structure retrieval

We solve the inverse problem of constraining Io’s internal structure using MCMC. We test two internal structure models with and without a magma ocean. A large parameter space is explored using the affine invariant ensemble sampler implemented in the open-source library, emcee^81^. Our full model input is given by the vector of input parameters given in Extended Data Table 3 for the magma ocean case and Extended Data Table 4 for the no magma ocean case. Extended Data Tables 3 and 4 also show the parameters of the prior probability distribution for each parameter.

The MCMC sampler is then run to obtain layer thicknesses and densities as well as rheologic parameters, which are used to generate synthetic observations of static gravity and complex-valued Love number *k*_2_. The synthetic observation vector **X** = [*C*_20_,*C*_22_ Re(*k*_2_),Im(*k*_2_)]^T^ is compared with the observed values **Y** and their covariance matrix **Σ** by computing the log-likelihood function $\log L=-\frac{1}{2}{({\bf{X}}-{\bf{Y}})}^{T}{{\boldsymbol{\Sigma }}}^{-1}({\bf{X}}-{\bf{Y}})$. The full covariance matrix can be constructed using the data provided in Extended Data Table 1 assuming Im(*k*_2_) is not correlated with other parameters. The log-likelihood function is used to explore the parameter space and determine the posterior distribution of internal structure model parameters. We also compute the libration amplitude *γ* for each step in the Markov chain. The posterior distribution of *γ* is shown in Extended Data Fig. 4. There is an overlap between the two probability distributions, but smaller libration amplitudes are possible for the no magma ocean case. Full posterior distributions are shown in Extended Data Figs. 5 and 6.

**Extended Data Fig. 7:**
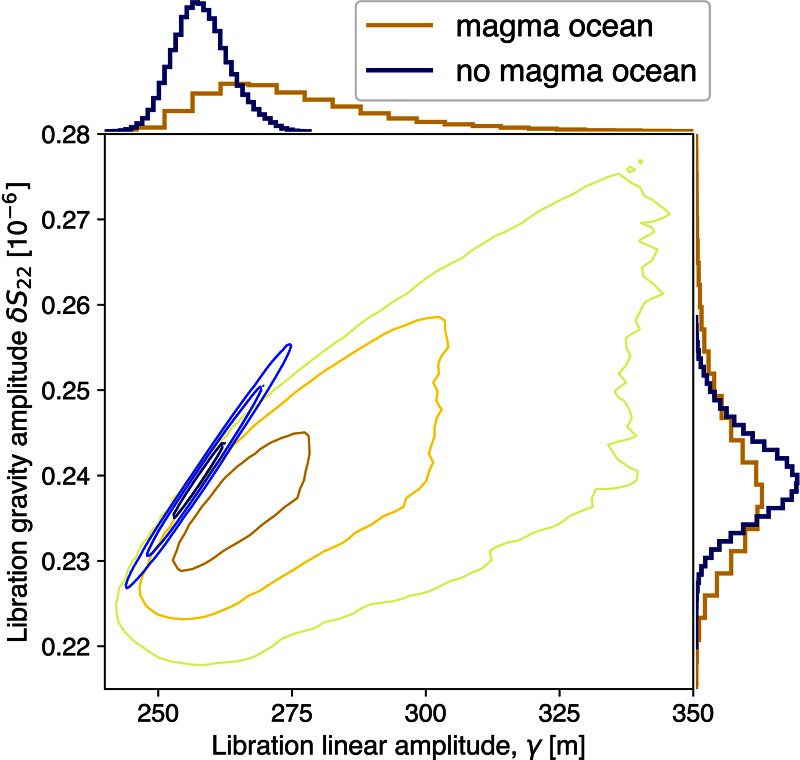
Posterior distribution of gravitational and linear libration amplitudes for the cases with and without a magma ocean. The vertical axis shows the gravitational libration amplitude and the horizontal axis shows the libration amplitude (1*σ*, 2*σ* and 3*σ* regions are shown).

Physical libration introduces an apparent time variability of *S*_22_. The amplitude of this variation is *δS*_22_, which we refer to as gravitational libration amplitude. *S*_22_ varies with respect to uniform rotation owing to the periodic oscillation of the outer solid shell and, in the magma ocean case, also the inner solid mantle. The shapes of the interfaces are assumed to be hydrostatic in our modelling. The posterior distributions for the linear and gravitational libration amplitudes are shown in Extended Data Fig. 7. For the no magma ocean case, there is nearly one-to-one correspondence between the two libration amplitudes. However, a wider range of libration amplitude combinations is possible if a magma ocean is present. Gravitational libration amplitudes are typically lower for the case with a magma ocean. Because the overlap between the two posterior distributions is minimal, future simultaneous measurements of the gravitational and linear libration amplitudes can be used to rule out even a deep magma ocean.

### Thickness of Io’s rigid lid

A rigid lid or elastic lithosphere of some thickness is necessary to support Io’s more than 100 towering mountains, some of which reach elevations 17 km above Io’s background plains^75^. These are widely interpreted as a product of Io’s heat-pipe volcanic cycle. In this Io’s copious volcanism reaches the surface through discrete conduits, but the continuous resurfacing causes downward advection of the cooled surface layers and increasing lateral compression at depth. This downward advection strongly suppresses radially outward heat conduction, away from volcanic centres^82^, stabilizing the required elastic lithosphere. Increasing compression eventually causes brittle failure at depth, forming thrust faults, which propagate upward through the lithosphere and breach the surface, creating the mountains^83^. These tectonic mountains themselves constrain the thickness of the elastic lithosphere that supports them. The minimum estimated thickness *d* is given by the tallest mountains (17 km). Mountains on Io are spaced on average about 600 km apart. If we suppose that all mountains initially form 10 km high, which implies approximately 15 km of horizontal displacement along a 30° inclined thrust ramp, then the horizontal strain implied is about 15/600 = 2.5%. This amount of horizontal strain is reached when a surface layer on Io is driven downward by about 46–50 km. A similar estimate of *d* ≲ 50 km was obtained previously^84^ by summing the total volume of Io’s mountains today and equating it to the volumetric strain at depth owing to faulting.

## Online content

Any methods, additional references, Nature Portfolio reporting summaries, source data, extended data, supplementary information, acknowledgements, peer review information; details of author contributions and competing interests; and statements of data and code availability are available at 10.1038/s41586-024-08442-5.

## Figures and Tables

**Extended Data Table 1:**
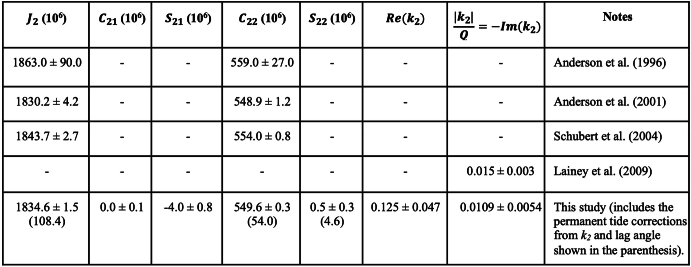
Recovered Io quadrupole gravity field and the tidal Love number *k*_2_

The spherical harmonic coefficients are unnormalized and uncertainties are formal 1*σ*. The reference radii used in some of the earlier analyses differ from ours; thus, the quadrupole harmonic values in this table have all been adjusted to the same reference radius of 1,829.4 km. In our estimation, the dissipation quality factor is directly estimated *Q* = 11.4 ± 3.6 (1*σ*) and combined with |*k*_2_| to compute the ratio |*k*_2_|/*Q* = −Im(*k*_2_). Our estimated *μ*_*i*_ is 5,959.8948 ± 0.0027 km^3^ s^−^^2^ (1*σ*). The correlations between Re(*k*_2_) and permanent tide correlated *J*_2_ and *C*_22_ are 0.0291 and 0.5120, respectively, whereas the correlation between permanent tide correlated *J*_2_ and *C*_22_ is 0.0026.

**Extended Data Table 2:**
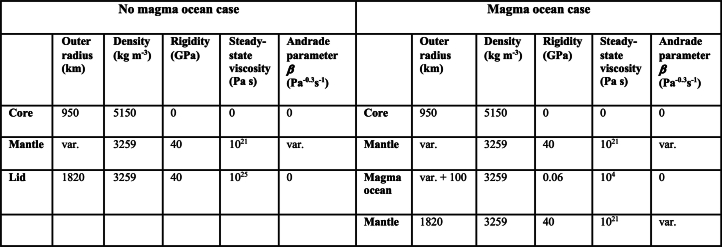
Tabulation of model parameters for no magma ocean and magma ocean cases

See text. var, variable. Our default magma ocean thickness is 100 km.

**Extended Data Table 3:**
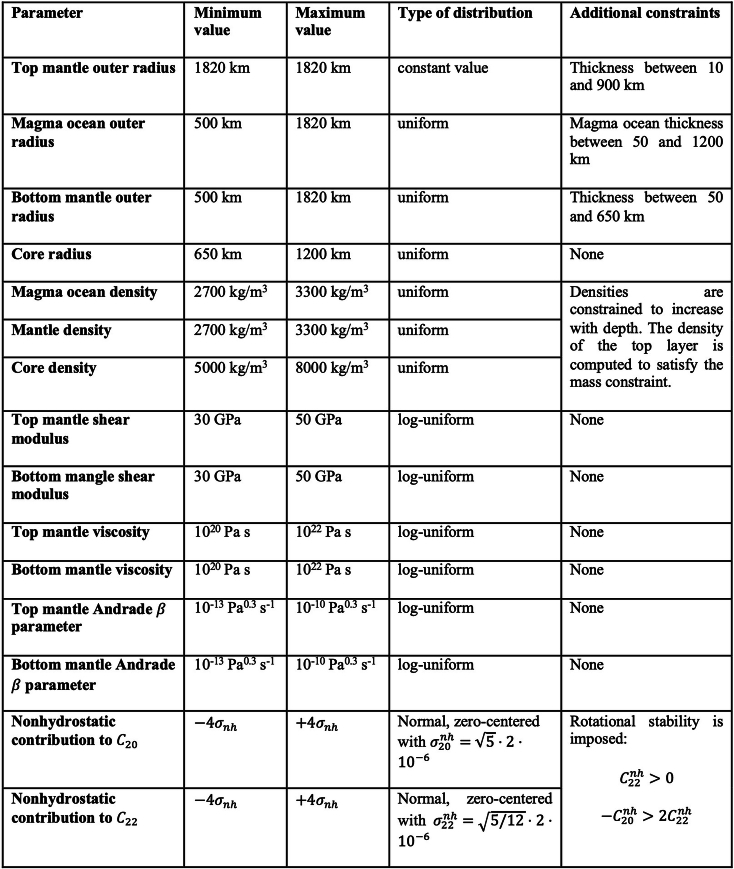
Full model input for the magma ocean case

Parameters of the prior probability distribution for the internal structure retrieval of the four-layer modelling including a magma ocean.

**Extended Data Table 4:**
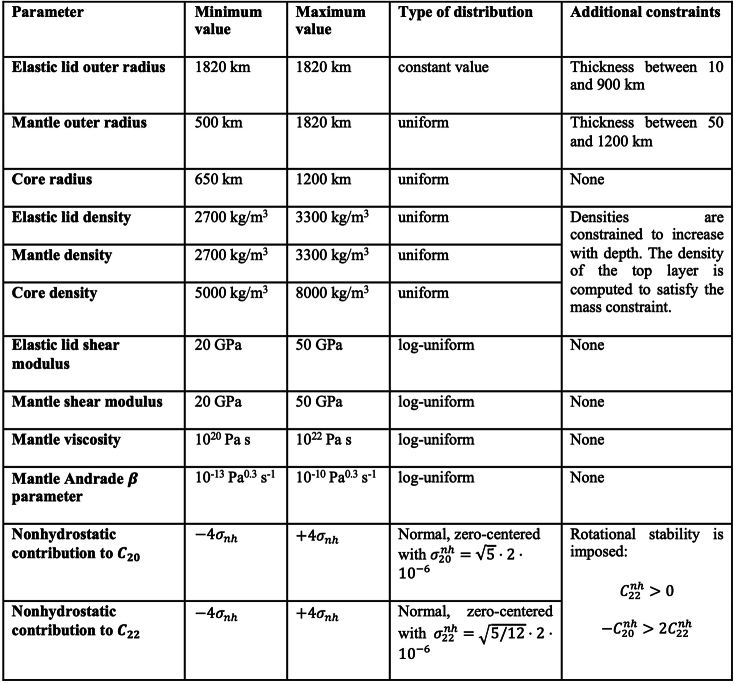
Full model input for the no magma ocean case

Parameters of the prior probability distribution for the internal structure retrieval of the three-layer modelling without a magma ocean.

## Data Availability

The Juno radio science data used in this research are publicly available through NASA’s Planetary Data System at https://atmos.nmsu.edu/PDS/data/jnogrv_1001/. Partial Galileo data are available through the NASA Planetary Data System at https://pds-ppi.igpp.ucla.edu/.

## Code availability

The results presented in this study can be reproduced using the MONTE software. The license for MONTE can be requested through https://montepy.jpl.nasa.gov/. The code that can reproduce our MCMC results is available on Zenodo at 10.5281/zenodo.14029354 (ref. ^85^).
